# Epidemiological Survey of Rotaviruses Responsible for Infantile Diarrhea by the Immunomolecular Technique in Cotonou (Benin, West Africa)

**DOI:** 10.1155/2018/3602967

**Published:** 2018-05-08

**Authors:** Jijoho Mischaël Michel Agbla, Annick Capo-Chichi, Alidéhou Jerrold Agbankpé, Tamègnon Victorien Dougnon, Anges William M. Yadouleton, Olivia Houngbégnon, Clément Glele-Kakai, George Enyimah Armah, Honoré Bankolé

**Affiliations:** ^1^National Health Laboratory, Ministry of Public Health, 01 P.O. Box, 418 Cotonou, Benin; ^2^Departmental Teaching Hospital Suru-Léré, Pediatric Service, 01 P.O. Box, 1643 Cotonou, Benin; ^3^Research Unit in Applied Microbiology and Pharmacology of Natural Substances, Research Laboratory in Applied Biology, Polytechnic School of Abomey-Calavi, University of Abomey-Calavi, 01 P.O. Box, 2009 Cotonou, Benin; ^4^Centre of Research in Entomology Cotonou, Ministry of Public Health, 01 P.O. Box, 418 Cotonou, Benin; ^5^Epidemiological Surveillance Service, Ministry of Public Health, 01 P.O. Box, 418 Cotonou, Benin; ^6^Noguchi Memorial Institute for Medical Research, University of Legon, P.O. Box LG 581, Legon, Accra, Ghana

## Abstract

Rotavirus remains the main causative agent of gastroenteritis in young children, in countries that have not yet introduced the vaccine. Benin, in order to implement the WHO recommendations, projects to introduce the rotavirus vaccine in 2018 as part of its Expanded Program on Immunization. But before the introduction of this vaccine, epidemiological data on rotavirus infections and rotavirus genotypes circulating in Benin should be available. The aim of this study is to generate epidemiological data on infantile rotavirus diarrhea in Benin. In order to determine the epidemiological characteristics and electrophoretypes of rotavirus responsible for gastroenteritis in diarrheic children aged 0 to 5 years, 186 stool samples were collected according to the WHO Rotavirus Laboratory Manual from March 2014 to February 2015 at Suru-Lere University Hospital Center. Detection of rotavirus antigen was performed by the ELISA test, followed by molecular characterization using polyacrylamide gel electrophoresis. 186 stool samples were analyzed for rotavirus, and seventy-three (39.2%) were found to be positive for rotavirus antigen by ELISA. Children aged 3 to 24 months were the most affected by rotavirus diarrhea in this study. Of the seventy-three children affected with rotavirus diarrhea, 27 (37%) had vomiting accompanied by dehydration and fever. Results based on electrophoresis showed that, among the 73 samples tested, 38 yielded typical rotavirus electrophoretic migration profiles.

## 1. Introduction

In developing countries, diarrhea is a major cause of infant morbidity and mortality [1]. Indeed, it is the second leading cause of death in children under 5 years of age, with 10% deaths each year [2]. Among the etiologic agents of diarrhea, viruses lead the way with 80% of cases. Among these viruses, rotaviruses are the most represented [3]. Globally, the rotavirus deaths in 2013 were estimated at 215,000 in children under five years of age [1]. According to the World Health Organization and Centers for Disease Control and Prevention (WHO/CDC) and the Global Burden of Disease Study (GBD), this estimate is, respectively, 215,757 and 122,322 in 2013 [4]. The largest number of rotavirus deaths occurred in sub-Saharan Africa, where the number ranged from 250,000 deaths in 2000 to 121,000 deaths in 2013 [1], with most cases being manifested as acute gastroenteritis.

Malnutrition, impaired individual and collective hygiene, and underutilization of oral rehydration solutions contribute to the severity of the clinical expression of infantile viral gastroenteritis, mainly in sub-Saharan Africa and Asia [5–7]. Fighting against rotavirus infection is a major concern for the World Health Organization for several years [1]. Improving water supply, promoting community sanitation, washing hands with soap, encouraging breast-feeding, and providing vitamin A supplements are preventive measures against rotavirus diseases [8]. Therapeutic interventions are mainly based on administration of zinc tablets and administration of reduced osmolality oral rehydration salts for the replacement of water losses. This emergency health care is often inaccessible or nonexistent in developing countries, which makes prevention of rotavirus by essential vaccination to save children's lives [9]. In 2009, the World Health Organization (WHO) recommended that all countries, and particularly those countries with high diarrhea mortality rates in children, introduce rotavirus vaccines into their national immunization programs [10]. By the end of 2014, >70 countries had introduced rotavirus vaccine into their routine immunization programs for children [1]. Several countries that have implemented routine childhood vaccination against rotavirus have documented a tremendous impact on severe diarrhea and rotavirus disease requiring hospitalization [11].

In Benin, according to statistics from the Demographic Health Survey, the infant mortality rate due to diarrhea remains very high. Although epidemiological data are available in relation to bacterial and parasitic diarrhea, this is not the case for viral diarrhea in general and particularly for rotavirus diarrhea where no data are available. In compliance with WHO recommendations for the integration of rotavirus vaccines into all national immunization programs, Benin through the National Agency for Immunization and Primary Health Care projects to introduce in 2018 the rotavirus vaccine in its full multiannual vaccination plan (2014–2018) [12]. The objective of this multiannual plan is to vaccinate at least 50% of the targets in 2018 to reduce morbidity and mortality associated with rotavirus infections [12]. But for better care or surveillance of rotavirus gastroenteritis, it was first necessary to have an upstream idea about the epidemiological data of rotavirus diarrhea in children in Benin.

The aims of this study are to determine the prevalence, the epidemiological characteristics, and the electrophoretypes of circulating rotavirus.

## 2. Methodology

This was a cross-sectional experimental study carried out from March 2014 to February 2015. Included in this study were children aged 0 to 5 years without distinction of sex and admitted to the Suru-Lere University Hospital Center due to gastroenteritis made of episodes of diarrhea. Excluded from this study were all children with bloody stools mainly due to other pathogens.

### 2.1. Sampling

The size of the sample chosen was determined by the following Schwartz formula:

(1) $$\begin{array}{c} N=\frac{t^{2}\left(p\left(1-p\right)\right)}{I^{2}}, \end{array}$$

where *N* is the sample size, *t* is the confidence level at 95% (=1, 96), *I* is the error margin (8%), and *p* is the prevalence (40%) according to the WHO [8].

At least a total of 144 samples are needed for this study. We collected 186 stool samples for the study. All cases of diarrhea that met the inclusion criteria were collected at the study period.

### 2.2. Sample Collection

According to the WHO Rotavirus Laboratory Manual, 5 ml or 5 g of stool samples was collected from children in clean 10 ml plastic containers. For liquid stool, pots were used to collect the samples, which are then transferred to the appropriate containers. The collection was made with the help of the children's parents. Stool collection has been done at least 48 hours after hospital admission of children to avoid nosocomial infections. These samples were then transported to the National Public Health Laboratory of Benin for analysis in a cooler containing cold accumulator with a prefilled identification code.

### 2.3. Analytical Methods

For each sample, 3 aliquots were taken and numbered 1 to 3. Aliquot no. 1 was used for the research of the VP6 antigen, the major protein of the intermediate capsid, by ELISA. The two others (nos. 2 and 3) after addition of 200 *μ*l drops of glycerol (on each) were stored in the freezer at −20°C for the determination of the electrophoretic profiles in the Reference Laboratory at Noguchi Memorial Institute for Medical Research, University of Legon, Ghana.

#### 2.3.1. Selection of Stool Samples Containing Rotavirus

The selection of stool samples containing the rotaviruses was based on the determination of the VP6 protein. The determination of the VP6 protein was carried out according to the ELISA technique.

*(1) Technical*. The ELISA test was carried out on various stool samples according to the method described in the ProSpecT Rotavirus kit marketed by Oxoid. The various ProSpecT Rotavirus kit reagents and the stool samples to be tested were taken out of the refrigerator 30 minutes before handling. A 10% suspension was prepared by adding 100 *μ*L of liquid stools or 0.1 g of solid stools or about the size of a pea to 900 *μ*L of the diluent supplied with the kit. The suspension was mixed with the vortex stirrer for 30–60 seconds and then centrifuged at 5000 rpm for 3 min. A volume of 100 *μ*L of supernatant from each diluted sample was added to the various microwells following a preestablished bench sheet. Positive control and negative control were always included in each set of tests. Then, 100 *μ*L of the conjugate was added to each microwell. The conjugate contains polyclonal antibodies specific to VP6 protein and conjugated to peroxidase. The plate was coated and incubated at 22°C for 60 ± 5 minutes. After incubation, the contents of the microwells were poured, where each microwell was washed with a wash buffer previously diluted to 1/10. This washing process was carried out 5 times in total. After the final wash, the plate was spilled and patted on absorbent paper to remove the last traces of wash buffer. After this washing, 100 *μ*L of the substrate was added to each microwell. The microwells were then incubated at 22°C for 10 minutes and received 100 *μ*L of stop solution. The reading step was performed within 30 minutes after the addition of the stop solution.

*(2) Reading*. The reading was taken from an ELISA reader at dual wavelengths 450 and 650 nm. The cleanliness of the bottom of the microwells was carefully checked before taking the reading. The calculation of the cutoff value was done by adding 0.200 absorbance units to the negative control value according to the manufacturer's recommendations and approved by the WHO reference laboratories.

*(3) Validation and Interpretation of Results*. The test has been validated if the value of the negative control is strictly less than 0.150 units of absorbance and that of the positive control is strictly greater than 0.500 units of absorbance. At the end of the reading, any sample of stools whose absorbance unit value is greater than the cutoff value is positive, and any sample of stools whose absorbance unit value is less than the cutoff value is declared as negative. Samples whose results gave values of absorbance units less than 0.010 units of absorbance of the cutoff value were considered equivocal. These samples were retested.

#### 2.3.2. Determination of the Electrophoretic Profile of Rotaviruses

All aliquots numbered 2 and 3, whose correspondents were ELISA positive, were transported (by air) in a triple package containing dry ice to Reference Laboratory at Noguchi Memorial Institute for Medical Research. Polyacrylamide gel electrophoresis (PAGE) was carried out on the faecal suspensions by the procedure described by Steele and Alexander [13]. The dsRNA genome was extracted using phenol-chloroform deproteinization and ethanol precipitation. Extracted viral genome from all ELISA rotavirus-positive samples was applied to separate lanes of the 10% polyacrylamide slab and electrophoresed for 18 hours at 100 V at room temperature using 1X Tris-glycine running buffer (5 mM Tris and 50 mM glycine) in the discontinuous buffer system. The dsRNA segments were visualized by silver staining according to the method described by Herring et al. [14].

#### 2.3.3. Data Analysis

The data were analyzed by the SPSS software version 21. The analysis was done at the 5% threshold in order to detect a possible comparison.

## 3. Results

The mean age obtained in the setting of this survey was 11 months with a more important representativeness (46.3%) at the age of 7 to 12 months. Of the 186 children, 55.4% were male (Table 1).

### 3.1. Distribution of the Patients' State according to Some Clinical Parameters

**Figure 1:**
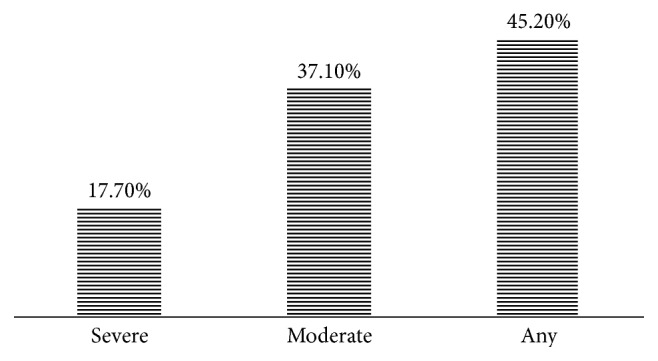
Distribution of study population according to dehydration state.

**Figure 2:**
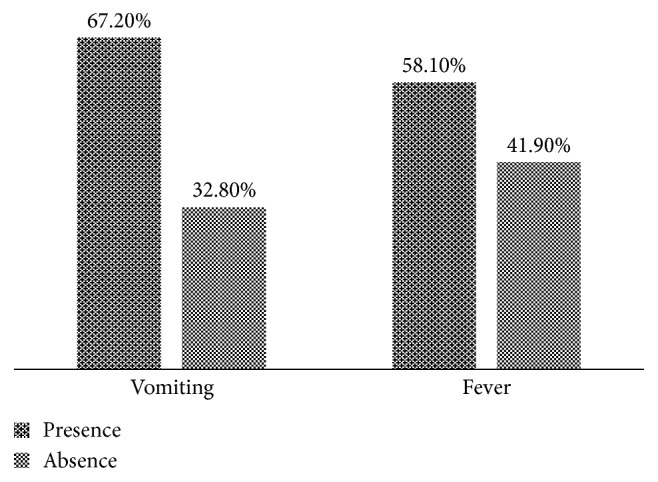
Frequencies of the patients according to the presence or absence of fever and vomiting.

With regard to the dehydration state of the patients, more than half of the patients were dehydrated (54.8%) and that to various degrees. 37.1% of the children were moderately dehydrated, followed by 17.7% of children severely dehydrated (Figure 1). More than half of the subjects had fever (T > 37.5°C) and vomited (Figure 2).

### 3.2. Result of Rotavirus Infection

A total of 186 diarrheic children were analyzed for rotavirus, and 73 (39.2%) were found to be positive for rotavirus antigen by ELISA.

#### 3.2.1. Distribution of Rotavirus Diarrhea by Month and Age

**Figure 3:**
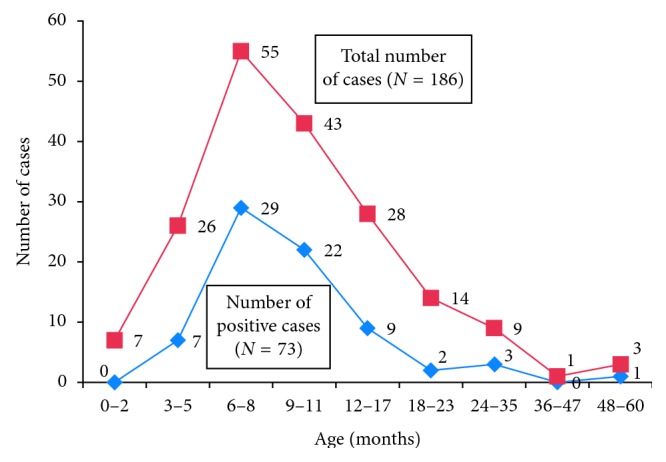
Distribution of rotavirus diarrhea according to age.

Rotavirus was isolated during all months of the year with peaks during the main dry season (January–February), the rainy season (June), and the small rainy season (September–October). However, there is no statistically significant difference (*p*=0.286) (Table 2). Children aged 0–3 months and 24–60 months were the least affected by rotavirus diarrhea in this study. This was not the case for those aged between 3 and 24 months. Statistical analysis showed a significant difference between the two groups of children and infection (*p* < 0.05) (Figure 3).

#### 3.2.2. Distribution of the Children Infected by Rotavirus according to Clinical Parameters

The detection of rotavirus by ELISA is significantly associated with more severe disease in children (*p*=0.015). Of the seventy-three children affected with rotavirus diarrhea, 38 (52.1%) had diarrhea with vomiting and severe dehydration (Table 3). This same proportion of children, in addition to diarrhea and vomiting, had fever (Table 4). There is a statistically significant difference (*p*=0.009; *p*=0.034) between these proportions. The symptoms of vomiting-dehydration, on the one hand, and vomiting-fever, on the other hand, are associated with the onset of rotavirus diarrhea. In addition, Table 5 shows that 37% (27/73) of children with rotavirus diarrhea had vomiting accompanied by dehydration and fever (*p*=0.015).

#### 3.2.3. Electrophoretic Profile: Polyacrylamide Gel Electrophoresis (PAGE)

**Figure 4:**
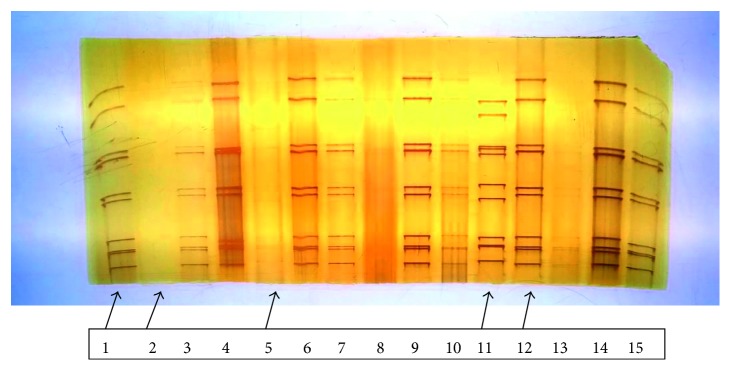
Electrophoretic profiles of rotavirus RNAs. (1) Positive; (2) negative; (5) positive faint; (11) short profile; (12) long profile.

All electrophoretypes obtained belong to group A of rotavirus characterized by the genomic distribution profile 4-2-3-2. Among the 73 rotavirus stool samples examined by polyacrylamide gel electrophoresis, 38 yielded typical rotavirus electrophoretic migration profiles while the remaining 35 isolates showed no profile. There is a predominance of long RNA electropherotype with 71% and 29% for short profile (Figure 4). In this hospital-based study, the long electrophoretic profile was the most prevalent rotaviruses in circulation. The long profile is much more observed (59.3%) in the age range of 7 to 12 months. The same is true for the short profile (72.7%) (Table 6). There is a predominance of the long profile with a percentage of 71% against 29% of short profile.

## 4. Discussion

Rotaviruses were found to be an important agent of diarrhea, leading to hospitalization in children less than 5 years of age in southern Benin. A 39.2% of diarrhea hospitalization was found to be associated with Group A rotavirus infection. The incidence of rotavirus is almost similar in developed and developing countries and varies from one country to another or even in one country from one region to another [15]. Numerous studies have shown the important role of rotaviruses as a cause of diarrhea in children in developed and developing countries, most of which have occurred in children under 5 years of age [16, 17]. This prevalence is similar to that in [18, 19, 20, 21], Kenya [22], and Jordan [23] in children of the same age. It is higher than that found in Gabon [24] and Angola [25].

Although our results show the presence of rotavirus infection throughout the year, high rates are recorded during the rainy season and the long dry season. This finding is identical to that reported in South Asia in a meta-analysis by Jagai et al. on the seasonality of rotavirus infection, and they found higher rates during the colder and dry months [26]. According to the WHO, rotavirus is isolated throughout the year in the subtropical zone and peaks often occur in dry seasons, unlike in developed countries where peaks occur in winter [8]. In regions with temperate climates, numerous studies have shown that rotavirus infections occur mainly during the cold winter season; this is the case of Iran [27], Europe [28], and the United States [29]. However, rotavirus distribution can vary greatly from year to year in subtropical countries [8]. This situation suggests taking into account local climatic variations for a better knowledge of the seasonal distribution.

About 79.45% (58/73) of children infected with rotavirus were aged 12 months or less with a peak between 6 and 8 months. The study conducted in Cameroon by Mbuh et al. reports 40% of cases between 7 and 9 months of age [30]. In Uganda, Nakawesi et al. reported that infected children were at an average age of 10 months [31]. In Nigeria, Jumaid et al. found a peak of rotavirus infection in children aged 7 to 12 months [20]. As we see, rotavirus infection mainly affects children under 2 years of age with peak incidence in children 6–11 months of age. This could be explained by the protective effect of maternal antibodies in children under 6 months of age as well as the development of natural immunity after repeated infections in children over 2 years of age [32, 33]. Since the breastfeeding rate begins to decrease after 6 months, Clemens points out that the protective effects of breastfeeding also seem to decrease with age [34]. The reduction in the number of cases after 24 months could be justified by the fact that these children have been in contact with the virus at least once and have therefore developed immunity.

Rotavirus infection in infants and young children can lead to severe diarrhea, dehydration, electrolyte imbalance, and metabolic acidosis [35]. The comparison between infants infected and noninfected with rotavirus shows high proportions of vomiting, dehydration, and fever in infected individuals, although the difference was not statistically significant. These observations are consistent with those reported in Nigeria [17, 36] and the Democratic Republic of Congo [37]. By contrast, Mukaratirwa et al. [38] in Zimbabwe found that vomiting varied significantly with rotavirus infection. The distributions of children according to the association of these signs were statistically significant (*p* < 0.05). The same results were obtained in Nigeria [20] and in the United States [39]. This could be explained by the fact that rotavirus would simultaneously cause at least two of these clinical signs associated with the presence of diarrhea [20, 39]. These facts justify the high admission rate and especially hospitalization observed.

Reports from different parts of the world have indicated electrotyping as a potential tool for studying the molecular epidemiology of human rotavirus infections [40]. In this study, the predominance of long profile over short one, irrespective of the zone of the country, is in accordance with the findings of other researchers, and it seems to be a norm [41–43]. There were more electrophoretic profiles observed in this present study than those reported previously in Nigeria [41, 43]. The mechanisms of generating extensive genomic diversity among human rotavirus strains are due to genetic reassortment [44], as genetic rearrangement occurs either naturally or probably due to imposition of pressure by the host immune system [45].

Twenty-five (34.25%) samples were ELISA positive but did not show any RNA profile on PAGE. This could perhaps be as a result of insufficient intact RNA in the specimens for PAGE to detect, as the sensitivity limit of PAGE stained by the silver nitrate is 3.4 ng of viral genomic RNA [14], or may be that the RNA might have been degenerated during storage due to constant power failure in the country.

This study has limitations, including the study area that only included patients from Suru-Lere Hospital. There is a lack of molecular identification of the different strains of rotavirus that exists in southern Benin, which is part of our research perspective. However, the strength of our study is certain. This is the first survey conducted in Benin on acute diarrhea that amply demonstrates the role of rotavirus in our environment. The results of this study will serve the health authorities in the short or medium term in their coherent decision-making on the adequate control of acute rotavirus diarrhea in southern Benin and, above all, have data for the trial phase in the context of the introduction of rotavirus vaccine into the vaccination program planned for the year 2018.

## 5. Conclusion

Rotavirus is present in the Cotonou city (southern Benin) and affects mostly children aged ≤12 months, during the dry and rainy seasons without distinction of sex, and leads quickly to diarrhea accompanied by vomiting, moderate/severe dehydration, and fever. Early and appropriate management will prevent deaths; environmental sanitation, hand washing, water intake, and especially rotavirus vaccination are the most effective preventive measures against rotavirus.

## Figures and Tables

**Table 1:** Distribution of study population by age and sex.

Age (months)	Sex		Effective
	M	F	
≤6	27 (14.5%)	28 (15.1%)	55 (29.6%)
7–12	52 (28.0%)	34 (18.3%)	86 (46.3%)
13–18	11 (5.9%)	11 (5.9%)	22 (11.8%)
19–24	7 (3.8%)	4 (2.1%)	11 (5.9%)
≥25	6 (3.2%)	6 (3.2%)	12 (6.4%)
Total	103 (55.4%)	83 (44.6%)	186 (100%)

**Table 2:** Distribution of rotavirus diarrhea according to months.

Months	Infected	Noninfected	Total	*χ* ^2^	df	*p* value
January	12 (36.4%)	21 (63.6%)	33	13.109	11	0.286
February	11 (42.3%)	15 (57.7%)	26			
March	4 (30.8%)	9 (69.2%)	13			
April	5 (55.6%)	4 (44.4%)	9			
May	2 (100.0%)	0 (0%)	2			
June	7 (50.0%)	7 (50.0%)	14			
July	3 (21.4%)	11 (78.6%)	14			
August	0 (0%)	3 (100.0%)	3			
September	10 (45.4%)	12 (54.5%)	22			
October	10 (47.6%)	11 (52.4%)	21			
November	3 (60.0%)	2 (40.0%)	5			
December	6 (25.0%)	18 (75.0%)	24			
Total	73 (39.2%)	113 (60.8%)	186			

**Table 3:** Distribution of children according to the state of dehydration associated with vomiting.

Dehydration + vomiting	Infected	Noninfected	*χ* ^2^	df	*p* value
Presence	38 (52.1%)	36 (31.9%)	6.730	1	0.009
Absence	35 (47.9%)	77 (68.1%)			
Total	73	113			

**Table 4:** Distribution of children according to the presence of vomiting associated with fever.

Vomiting + fever	Infected	Noninfected	*χ* ^2^	df	*p* value
Presence	38 (52.05%)	41 (36.28%)	4.415	1	0.034
Absence	35 (47.95%)	72 (63.72%)			
Total	73	113			

**Table 5:** Distribution of children according to the state of dehydration associated with vomiting and fever.

Dehydration + vomiting + fever	Infected	Noninfected	*χ* ^2^	df	*p* value
Presence	27 (37.0%)	20 (17.7%)	5.87	1	0.015
Absence	46 (63.0%)	93 (82.3%)			
Total	73	113			

**Table 6:** Distribution of readable profiles according to age groups.

Age (months)	Readable profile			
	Long	%	Short	%
≤6	7	25.9	1	9.1
7–12	16	59.3	8	72.7
13–18	1	3.7	1	9.1
19–24	1	3.7	1	9.1
≥25	2	7.4	0	0
Total	27	100	11	100
